# The biology of exosomes and exosomal non-coding RNAs in cardiovascular diseases

**DOI:** 10.3389/fphar.2025.1529375

**Published:** 2025-05-26

**Authors:** Lu Gan, Xiaofei Guo, Shichao Dong, Chuan Sun

**Affiliations:** ^1^ Department of Pharmacy, Children’s Hospital of Nanjing Medical University, Nanjing, Jiangsu, China; ^2^ Department of Pharmacy, The Second Affiliated Hospital of Harbin Medical University, Harbin, Heilongjiang, China; ^3^ Department of Pharmacy, The Second Hospital of Tianjin Medical University, Tianjin, China

**Keywords:** exosomes, non-coding RNAs, cardiovascular diseases, biomarker, intercellular delivery systems

## Abstract

Cardiovascular diseases (CVDs) remain the leading cause of death worldwide, both in developed and developing countries. Despite the implementation of various measures in clinical practice that have shown certain curative effects, poor prognosis and irreversible pathological cardiac remodeling continue to limit the therapeutic effect of CVDs. There are still many new mechanisms worth exploring for the regulation of CVDs. Previous studies have highlighted the potential applicability of exosomes in CVDs, and significant research has been conducted in this area. In this review, we summarize the physiological mechanisms of exosomes and the basic research achievements in regulating CVDs via exosomal non-coding RNAs. We also discuss the limitations and prospects of exosome application in CVD treatment.

## Introduction

Exosomes are small vesicles with an average diameter of approximately 100 nm and a lipid bilayer structure. They play a crucial role in intercellular communication and help maintain homeostasis in multicellular systems (Raposo and Stoorvogel, 2013; Hessvik et al., 2016). Exosomes are released by various cell types and can be detected in multiple biological fluids, including plasma, urine, saliva, and pericardial effusions (Michael et al., 2010; Kuosmanen et al., 2015). There is growing evidence to suggest that exosomes play important biological roles in both physiological and pathological conditions. These roles include influencing immune modulation, tumor invasion, and processes related to regeneration and degeneration (Bianco et al., 2007; Cai et al., 2012; Ma et al., 2017). Exosomes perform these functions by interacting with receptors on the surface of the recipient cell to deliver biomolecules such as lipids, proteins, messenger RNA (mRNA), and non-coding RNA (ncRNA) to the recipient cell. It is worth noting that ncRNAs are a component of exosomes and have attracted particular attention. In recent years, the scientific community has focused on non-coding RNAs. High-throughput RNA sequencing analyses of the genome have shown that more than 70% of the human genome is transcribed, but only 2% can encode proteins (Djebali et al., 2012). Previously, RNA molecules known as “junk” have been shown to have formed a large family with multiple functions. The non-coding RNA (ncRNAs) family consists mainly of microRNAs (miRNAs), long non-coding RNAs (lncRNAs), and circular RNA (circRNAs), which have received increasing attention from the cardiac community due to their ability to regulate the physiological and pathological processes of disease. In addition, certain non-coding RNAs exhibit stability in the blood and are markedly expressed to represent different disease states, suggesting that they may play a role as important disease biomarkers.

Cardiovascular diseases (CVDs) are the most common cause of morbidity and mortality worldwide, and their incidence is expected to increase in the coming years (Virani et al., 2021). Cardiovascular disease is the main cause of premature death in Chinese people, placing a heavy burden on public health (Liu et al., 2024a). Although new drugs have gradually entered clinical application in recent years, the progress of non-drug treatment methods such as intervention has provided a broader scope for the treatment of CVDs (Roth et al., 2020). However, the challenges of regenerating cardiomyocytes, reversing cardiomyocyte death, and addressing the side effects of invasive treatment modalities limit the reduction and recovery of the degree of cardiac decline. Therefore, reducing myocardial damage, inhibiting myocardial remodeling, promoting cardiomyocyte regeneration, and finding effective ways to prevent cardiovascular diseases are still urgent problems in the treatment of cardiovascular diseases. Recent studies have revealed that exosomes play a significant role in the regulation of cardiac function. These findings not only enhance our comprehension of the pathogenesis of CVDs but also pave the way for novel therapeutic approaches to combat CVDs.

Here, we first describe the biosynthesis, loading, and release of exosomes. We then focused on the therapeutic potential of extracellular vesicles loaded with different non-coding RNAs in cardiovascular disease. Finally, we provide an innovative summary and overview of current research advances and extended applications of extracellular vesicles in tissue engineering and clinical trials. We believe that at this stage, the advancement of extracellular vesicles from the laboratory to clinical practice still faces daunting challenges, requiring further exploration and interdisciplinary collaboration with fields such as materials science and industrial manufacturing. We believe that extracellular vesicles, as rapidly emerging players in regenerative medicine, offer new perspectives on the diagnosis, treatment, and prognosis of cardiovascular diseases.

### Biology of exosomes

In the late 1960s, the discovery of membrane-enclosed structures in the extracellular space, smaller than mammalian cells, provided additional understanding in this field. These structures were named extracellular vesicles (EVs). These nanosized vesicles (30–5000 nm in diameter), enclosed by a phospholipid bilayer, carry diverse bioactive cargo including proteins, genetic materials, and lipid components capable of regulating recipient cell activities through intercellular communication (Yanez-Mo et al., 2015). EVs are categorized into three distinct classes according to their formation pathways: exosomes, shed microvesicles, and apoptotic remnants, among which exosomal biogenesis initiates with plasma membrane invagination that generates early endosomal structures (Marar et al., 2021). These compartments subsequently develop into multivesicular bodies through intracellular membrane budding events, ultimately releasing exosomes via vesicle fusion with the cell membrane. During the past few years, much effort has been devoted to the research of exosomes, in terms of identifying the molecular composition, elucidating the mechanisms and regulations of biogenesis, and characterizing the functions in a variety of physiological and pathological ways. The latter two types of EVs are secreted by the plasma membrane of the cells. We will not go into details here.

**FIGURE 1:**
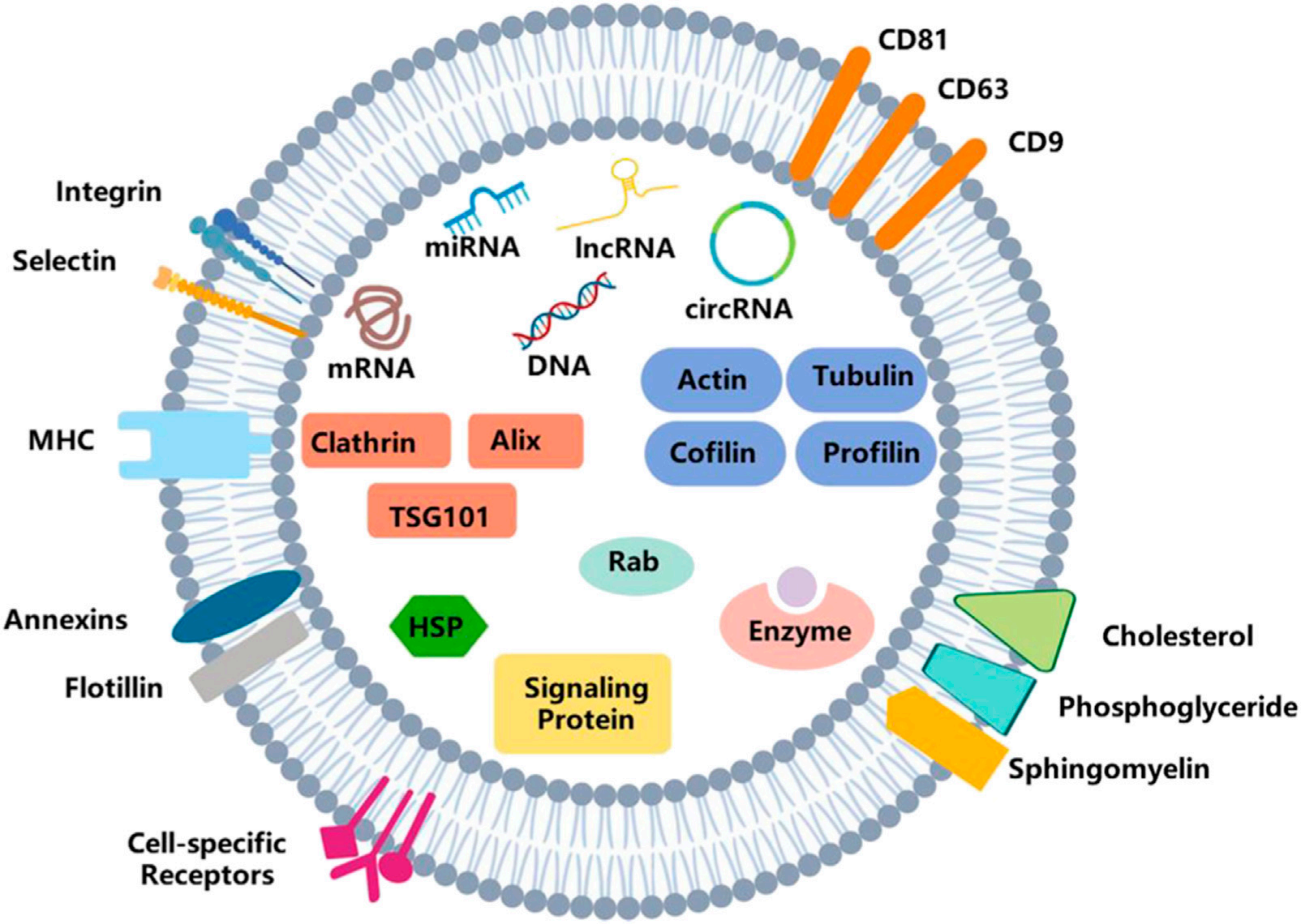
Structure of exosomes. Exosomes are vesicles with a double-membrane structure. The phospholipid bilayer is embedded with transmembrane proteins, lipid rafts, immune-regulatory molecules and receptors, etc. It also consists of DNA, RNA, enzymes, proteins, and other bioactive molecules.

Proteomics analyses have been performed on exosomes purified from different body fluids (Llorente et al., 2013; Pocsfalvi et al., 2016; Greening et al., 2017), and endosomal sorting complex required for transport (ESCRT) and tetraspanins proteins are identified (Larios et al., 2020). The former is known to play a vital role in cargo sorting and the formation of intraluminal vesicles (ILVs) (Schmidt and Teis, 2012; Kalra et al., 2016; Juan and Furthauer, 2018), while the latter is the fingerprint protein of exosomes (Andreu and Yanez-Mo, 2014; Kowal et al., 2014). Knowledge of the protein composition of exosomes can give us clues about the mechanism involved in their release; some functional proteins are also authenticated, including actin, annexins, tumor susceptibility gene 101 (TSG101), fibronectin-1 and vesicle-associated membrane protein 8, which participate in cellular movement, assembly, organization, and morphology. Regulatory ncRNAs are also important inclusions that alter gene expressions in target cells at the epigenetic level (Valadi et al., 2007; Yuan et al., 2009). In addition, ribosomal RNAs, transfer RNAs, and some different DNAs have been detected in exosomes in the past few years (Schuller et al., 2018). However, it is challenging to identify all these molecules. Some of them involved in this research are not necessarily incorporated into exosomes. The summary diagram of exosomes is shown in Figure 1.

### Exosome biogenesis

**FIGURE 2:**
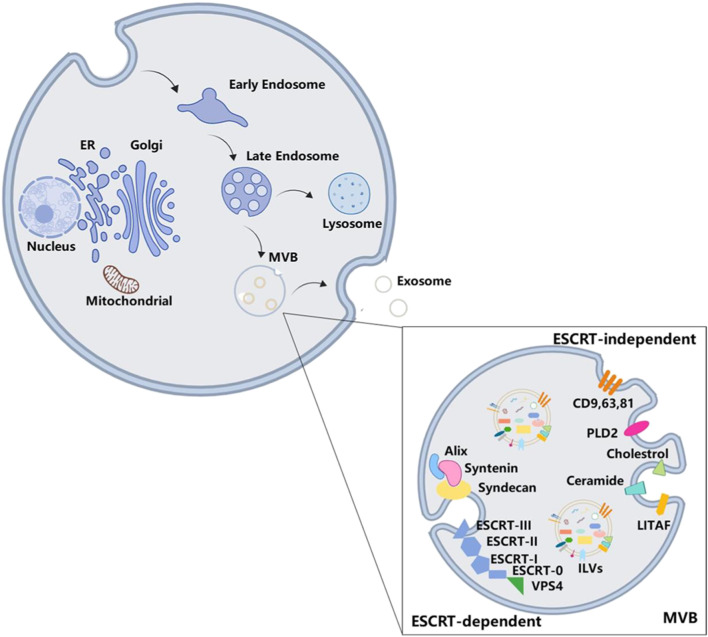
Biogenesis of exosomes. The formation of MVBs goes through intracellular endosomes inward budding inside, early endosomes, and late endosomes. Exosome is one of the final destinations of MVBs. There are two ways of exosome formation: ESCRT-dependent pathway and ESCRT-independent pathway.

Exosomes are first discovered in the maturing mammalian reticulocyte (Thery et al., 2002), and their origin is more complicated. EVs, which contain nuclear materials, cellular organelles, membrane components, and cytosolic contents (Elmore, 2007), originate from intracellular endosomes through inward budding of the endosomal membrane. During this process, early endosomes mature into late endosomes, which subsequently form multivesicular bodies (MVBs). Subsequently, ILVs are shaped and accumulated in MVBs, cytosolic proteins, and nucleic acid molecules and are packaged into them (Raposo and Stoorvogel, 2013). ILVs are the pre-exosomes. This process is shown in Figure 2.

Deeper insights into the protein composition of exosomes are necessary to fully understand the mechanism involved in exosome biogenesis. The ESCRT family comprises approximately 30 proteins, which are assembled into five functionally distinct subcomplexes: ESCRT-0 is responsible for cargo clustering in a ubiquitin-dependent manner; ESCRT-I/II are used to induce bud formation; ESCRT-III actuates vesicle fracture; and other accessory proteins, especially VPS4 ATPase, mediate the final membrane rupture and recycle of ESCRT (Vingtdeux et al., 2012; Henne et al., 2013).

During the formation of ILVs, endosomal proteins are recombined with abundant tetraspanins, including CD9, CD63, and CD81 (Akers et al., 2013; Kowal et al., 2016; Verweij et al., 2018). When ESCRT 0 combines with phosphatidylinositol 3-phosphate, ESCRT-1 is recruited into the endosomal membrane and subsequently forms the ESCRT 0/I complex. Next, ESCRT-I recruits ESCRT-II to induce oligomerization of the ESCRT-III complex (Anand et al., 2019), while cytosolic proteins are enriched in ILVs during endosomal membrane invagination to form ILVs in MVBs (Vingtdeux et al., 2012; Colombo et al., 2014; Villarroya-Beltri et al., 2014; Kalra et al., 2016). In addition, the Alix/syntenin/syndecan axis is also required for exosome formation. Through protein motifs, Alix interacts with the cytoplasmic adapter protein syntenin to facilitate budding by the ESCRT-dependent pathway (Baietti et al., 2012). The PDZ domain of syntenin including PSD95/Dlg/ZO-1 successively binds to the cytoplasmic tails of transmembrane heparan sulfate proteoglycan syndecan. This complex acts as a hub bridging exosomal cargo selection and ILV formation.

When the function of the ESCRT is destroyed, it does not inhibit MVB formation (Stuffers et al., 2009), suggesting that some fungible mechanisms could be considered for it, namely, the ESCRT-independent pathways. Tetraspanins enriched in exosomes are involved in ESCRT-independent exosome formation (Hessvik and Llorente, 2018). Previous studies have demonstrated that tetraspanins can play a role in the biogenesis of exosomes by affecting the exosomes’ release or changing their composition (Chairoungdua et al., 2010; Nazarenko et al., 2010; Hurwitz et al., 2016). Another protein, called lipopolysaccharide-induced TNF factor, interferes with the formation of MVBs when they are dysfunctional (Zhu et al., 2013). In addition to proteins, lipids have also been proven to be involved in the formation of exosomes. The conversion of sphingomyelin to ceramide is catalyzed by sphingomyelinase on the endosomal membrane, which is also a necessary process for the formation of ILVs and MVBs (Cheng et al., 2018). However, phospholipase D2, an inducer of the small GTPase ADP ribosylation factor 6, has been shown to regulate the formation of ILVs and participate in exosome biogenesis (Ghossoub et al., 2014). The biogenesis of exosomes has often been divided into two mechanisms that are dependent or not dependent on ESCRT, but these two regulation pathways might not be relatively isolated. Blocking ceramide conversion would decrease the expression level of TSG101, which belongs to ESCRT-0 (Xia Y. et al., 2020), meaning that there is a potential correlation between the two mechanisms.

### Sorting cargo into exosomes

**FIGURE 3:**
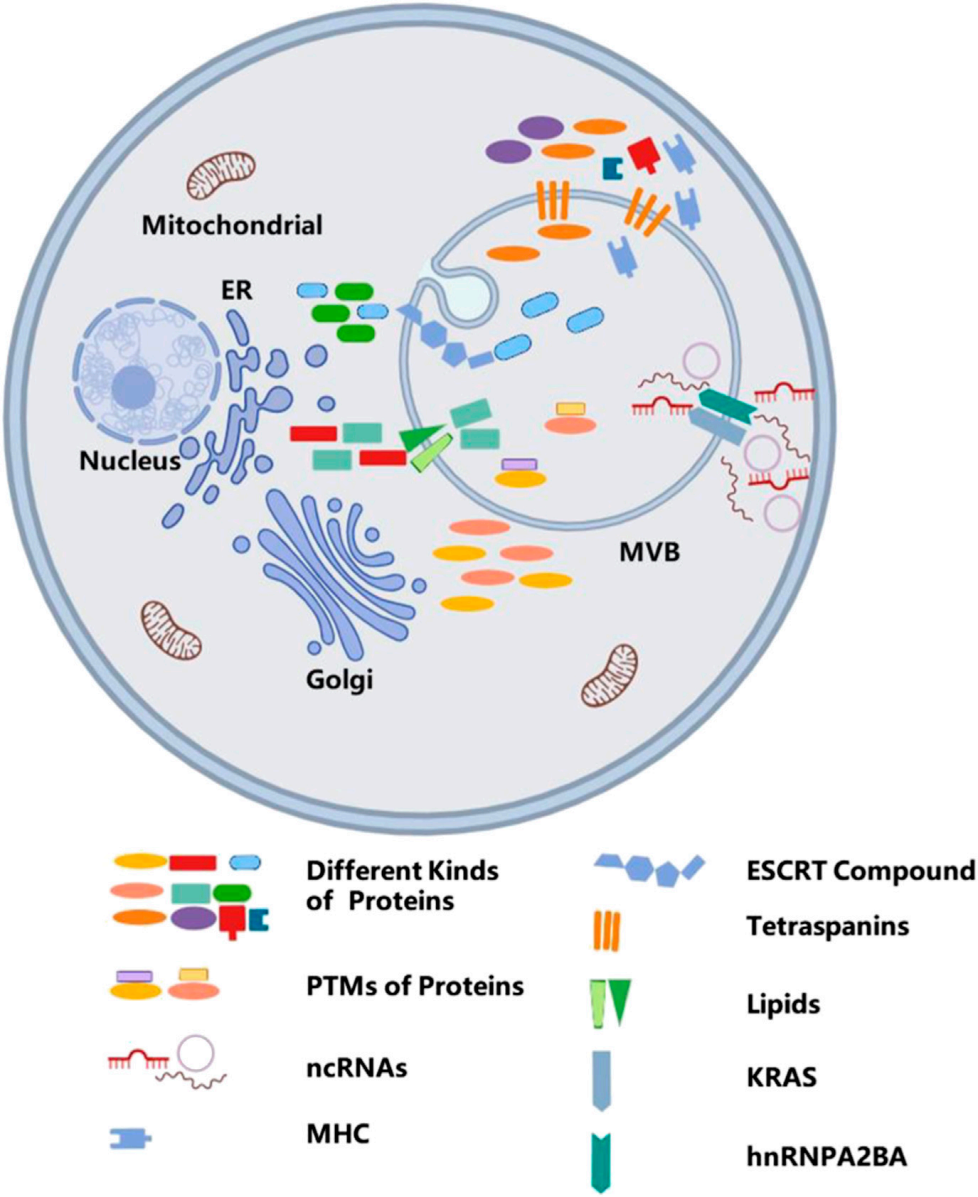
The mechanism of sorting cargos into exosomes. Sorting various bioactive molecules into MVBs is a complex process, and different molecules embedded in the phospholipid bilayer, such as ESCRT compound, tetraspanins, lipids, and KRAS, take part in sorting of different cargos. In addition, post-translational modifications also participate in sorting cargos.

MVBs have various sub-populations. Some are labeled with EGF and its receptor. Albeit with the same morphology, it is still different from the vacuole, which is marked with lysobisphosphatic acid, a late endosomal marker (Kobayashi et al., 2001). In addition, exosomes secreted by one polarized cell may have different compositions (van Niel et al., 2001; Tauro et al., 2013; Chen et al., 2016), which may also indicate the multiformity of MVBs. With regard to their different contents (Yuan et al., 2018), exosomes play various functions. Tumor-secreted exosomes have an essential role in the growth and metastatic evolution of primary tumors (Melo et al., 2014; Steinbichler et al., 2017). However, cardiomyocyte-derived exosomes mediate cell–cell communication and regulate cardiac function (Kuo et al., 2019). Above all, exosomes are enriched in heterogeneous components, suggesting that there is a specific mechanism to select certain molecules to enter the exosomes, and this process is not considered to be a random event. This process is shown in Figure 3.

Current studies show that proteins, lipids, and RNAs can be secreted into exosomes. The classification of cargo proteins sorted into exosomes depends on ESCRTs, tetraspanins, and lipids. As mentioned above, ESCRT mechanism proteins are implicated in the biogenesis of exosomes, whereas its components such as Alix and TSG101 also take part in the process of selectively packaging these proteins into exosomes.

In addition, post-translational modifications have been proven to control the selective mechanism of protein cargo sorting by regulating the subcellular localization, structure, and function of the proteins (Telekawa et al., 2018; Nasiri Kenari et al., 2019). Ubiquitination is one of the most important post-translational modifications for sorting of proteins into exosomes. Exosomes also contain a large number of ubiquitinated proteins (Buschow et al., 2005; Hessvik et al., 2016), which may be sorted into exosomes and induce higher levels of ubiquitin in exosomes (Smith et al., 2015; Cheng and Schorey, 2016). The composition of ESCRT is considered to select the mono-ubiquitinated transmembrane cargos and load them into ILVs (Villarroya-Beltri et al., 2014; Hilscher et al., 2016). Mono-ubiquitination of cargos avoids the binding of ubiquitination cargos and controls the sorting of certain proteins into MVBs (Henne et al., 2011). However, protein ubiquitination becomes a barrier under some circumstances. For instance, the sorting privilege of major histocompatibility complex II does not dependent on ubiquitination but requires the help of tetraspanin CD9 (Buschow et al., 2009; Gauvreau et al., 2009). In addition to ubiquitination, SUMOylation, ISGylation, phosphorylation, glycosylation, citrullination, myristoylation, oxidation, WW domain, and coiled-coil domain are also post-translational modifications and take part in regulating cargo sorting (Thomas et al., 2009; Poller et al., 2018; Telekawa et al., 2018; Ageta and Tsuchida, 2019; Carnino et al., 2020).

Exosome-derived RNAs include mRNAs and ncRNAs, such as microRNAs (miRNAs), long non-coding RNA (lncRNAs), circular RNAs (circRNAs), and ribosomal RNAs (Yang et al., 2017; Li Y. et al., 2019; Wang et al., 2019a). Sorting ncRNAs into exosomes may have concentration-dependent characteristics and could also be affected by competition with targets (Li et al., 2018). KRAS also play important roles in the classification of ncRNAs into exosomes. Compared to wild-type cells, mutations in KRAS would induce accumulation of certain ncRNAs and distinct ncRNA sorting (Cha et al., 2015). Furthermore, the expression of exosomal Argonaute 2, a component of the RNA-induced silencing complex, is downregulated, and its location in MVBs is also influenced in KRAS mutant cells (McKenzie et al., 2016), but it will be reversed by the activity of mitogen-activated protein kinase kinases I and II.

Compared to their parent cells, exosomes have been demonstrated to be enriched in cholesterol, sphingomyelin, and glycosphingolipids (Record et al., 2014; Skotland et al., 2017). This finding indicates exosomal membranes contain lipid rafts. Studies have shown that the conversion of proteins or molecules into lipid rafts may facilitate their assembly into early endosomes, suggesting lipid rafts play a regulator role in sorting exosomal cargos (Chakraborty and Jana, 2015). For example, continuous activation of G protein-coupled sphingosine 1-phosphate receptors, which are located on the MVB membrane, via sphingosine 1-phosphate, is essential for CD63, CD81, and flotillin sorting into ILVs destined for exosome release (Kajimoto et al., 2013). It is especially intriguing to inhibit S1P signaling to reduce ILV formation that is not affected by sorting molecules into exosomes. In addition, lipids also have a pleiotropic effect. By recruiting ESCRT components with sorting functions, phosphoinositides and lipid signaling intermediates can indirectly perform the sorting functions (Raiborg et al., 2001). In addition to participating in the formation of exosomes, the presence of lipids is also crucial for their uptake. The lipid composition of extracellular vesicle membranes has a significant impact on their morphology, stability, membrane fluidity, and charge characteristics. Increasing the lipid content of extracellular vesicles can enhance the rigidity of the extracellular vesicle membrane and regulate fusion stability with the target cell membrane (Simbari et al., 2016). The changes in the proportion of phospholipids in exosomes, such as phosphatidylserine and phosphatidylcholine, can affect the loading of membrane surface charges, affect their interaction with target cell membranes, and thereby regulate the recognition and uptake of exosomes by target cells (Ou et al., 2023). The lipid raft region rich in sphingolipids and cholesterol promotes lipid raft-dependent endocytosis pathways, which may enhance the delivery localization efficiency of targeted drugs, especially to tumor and inflammatory areas. Cholesterol-rich membranes in extracellular vesicles can delay their fusion with intracellular lysosomes or endosomes, thereby prolonging intracellular migration time and potentially isolating drugs from intracellular degradation, achieving sustained release from drug target sites (Joshi et al., 2020). A study has shown that, Onpattro, the first RNAi therapy strategy using lipid nanoparticle platforms can effectively deliver siRNA or miRNA into target cells. This strategy includes cationic lipids, cationic liposomes, and cationic polymers (Zheng et al., 2019). Another study found that cholesterol-rich extracellular vesicles can easily enter cancer cells through membrane fusion, achieving direct cytoplasmic delivery of goods. This type of extracellular vesicles may achieve better delivery efficiency compared to lipid nanoparticles (Zhuo et al., 2024).

### Secreting exosomes into the extracellular space

The fate of MVBs can either be fusion with lysosomes for degradation or transportation to the plasma membrane to release exosomes. The interferon-stimulated gene product 15 can be covalently linked to TSG101. ISGylation of TSG101 induced MVB co-localization with lysosomes and decreased exosome secretion (Villarroya-Beltri et al., 2016; Jimenez Fernandez et al., 2019). Recent studies indicate that exosome secretion is primarily regulated by the Rab family and soluble N-ethylmaleimide-sensitive factor attachment protein receptors.

The proteomic analysis validates the enrichment of Rab family members in exosomes. Rab proteins are a family of small GTPases that regulate the progression of intracellular vesicular trafficking, including budding, vesicle mobility through the cytoskeleton, or their location on the plasma membrane (Stenmark, 2009). These findings suggest that the Rab family may play roles in exosome secretion. Rab11 is the first Rab GTPase identified to be involved in exosome secretion. Rab35 is also necessary for the release of exosomes, which can control the membrane docking process (Yang et al., 2019). Downregulating Rab7 expression promotes secretion of ovarian cancer cell-derived exosomes in hypoxia. Different subtypes of Rab proteins may function in different cells. In Hela-CIITA cells, inhibition of Rab5A, Rab9A, and Rab2B could decrease exosome secretion, but Rab11 and Rab7 do not (Kowal et al., 2014). In addition, Rab27A and Rab27B have been proven to be involved in this process as they can recruit MVBs to the plasma membrane (Koh and Song, 2019).

These Rab proteins play a role in exosome secretion, which is necessary to recruit MVBs to the plasma membrane. However, the fusion of MVBs with the plasma membrane is also a crucial step for secreting exosomes into the extracellular environment. After docking the two intracellular compartments, soluble N-ethylmaleimide-sensitive fusion attachment protein receptors constitute the major machinery for lipid bilayer fusion and are also involved in intercellular vesicular trafficking. For instance, the consumption of SNAP23 and syntaxin-4 inhibits the fusion of MVBs with the plasma membrane in individual cells. On the contrary, enhancing the phosphorylation of Ser110 on SNAP23 may facilitate membrane fusion and secretion of exosomes (Verweij et al., 2018). There are studies demonstrating that lncRNA HOTAIR promotes membrane fusion by modulating the co-localization of VAMP3 and SNAP23 (Yang et al., 2019). Interestingly, Munc13-4, a Ca2+-dependent SNAP receptor and Rab-binding protein, uses a Rab11-dependent pathway that plays a role in membrane fusion (Messenger et al., 2018), suggesting that Rab family proteins may also be involved in this process.

**FIGURE 4:**
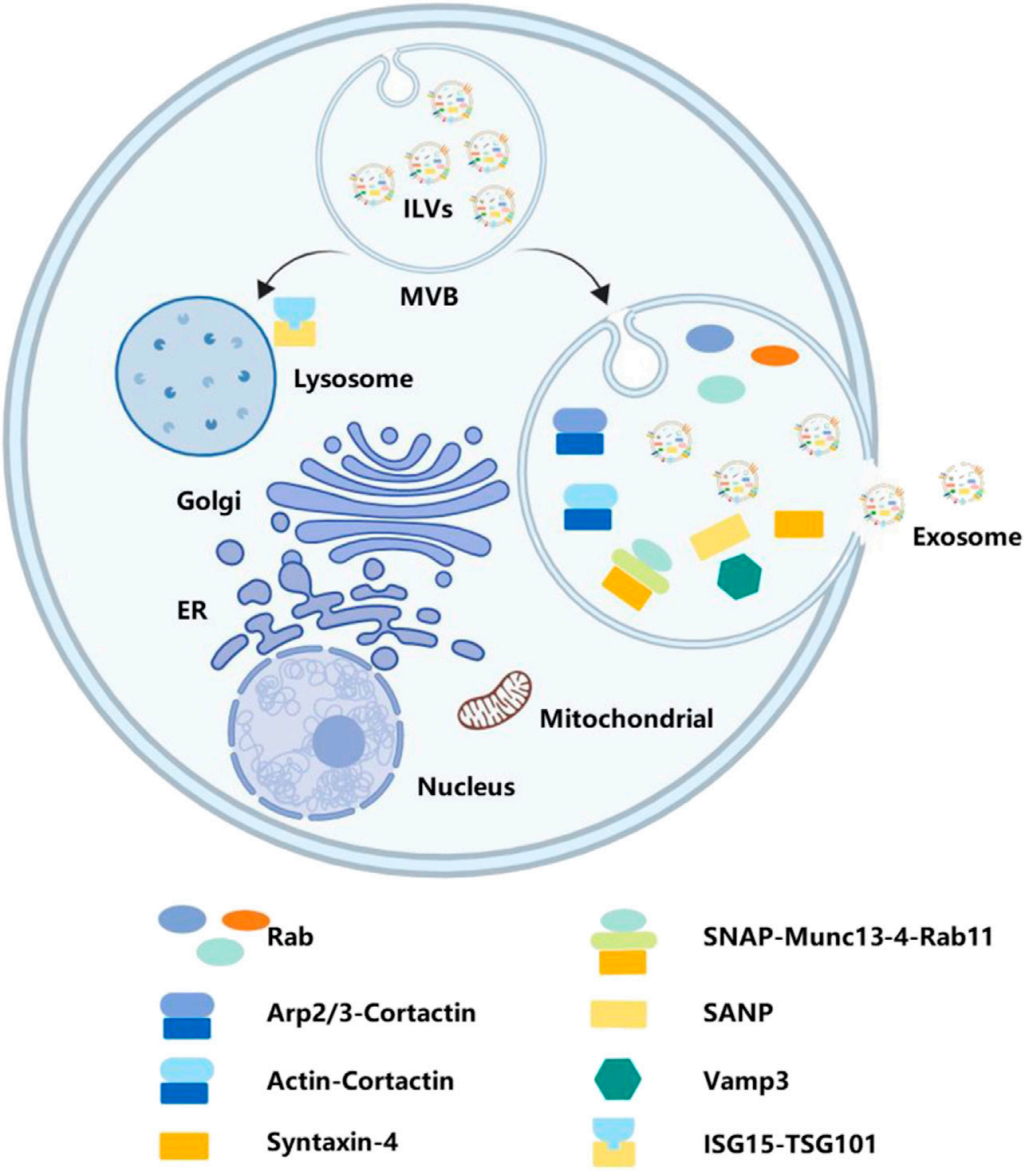
Secreting exosomes into extracellular space. Secreting exosomes into the extracellular space requires MVBs to fuse with the plasma membrane. Various bioactive molecules, including Rab, SANP, Vamp3, and TSG101, have play significant roles in this process.

Furthermore, the interferon-stimulated gene product 15 can be covalently linked to TSG101. ISGylation of TSG101 induced MVB co-localization with lysosomes and decreased exosome secretion (Qiao et al., 2019; Tang C. et al., 2023). Cortactin has also been confirmed to take part in the release of exosomes. By binding to the branched actin nucleating Arp2/3 complex and actin filaments, cortactin can promote exosome secretion (Sinha et al., 2016). In pancreatic cancer cell lines, the hypo-phosphorylation of cortactin induced by inhibiting PRKD1 expression can increase MVB release (Armacki et al., 2020). This process is shown in Figure 4.

### The role of exosomes in regulating CVDs

Cells play their biological functions by exchanging messages via direct interaction or secreting signaling molecules. Exosomes are probably one of the most important cell mediators which can interact among various cells and exert regulatory roles by transmitting exosomal cargos to target cells (Wortzel et al., 2019). Signaling molecules derived from exosomes, either protective information or detrimental information, are involved in regulating various CVDs, such as hypertrophy, cardiac remodeling, arrhythmia, and cardiomyopathy (Ibrahim and Marban, 2016). NcRNAs have been demonstrated to be important regulatory molecules of CVDs. In addition, current studies also indicate that exosomes take part in regulating CVDs via these molecules. Here, we primarily summarize relative research achievements of exosomes regulating CVDs via ncRNAs.

### Exosomes serve regulatory function via microRNAs

MicroRNAs, approximately 22 nucleotides, are the first ncRNAs to be studied in depth. By binding to the 3′ UTR, miRNAs can carry out negative roles in gene expression, such as controlling chromosomes, modification, and inhibiting transcription (Gu et al., 2009; Khalil et al., 2009). Yet, there is mounting evidence to suggest that miRNAs, which have been demonstrated to play significant roles in CVDs, are also involved in the process of exosomes regulating CVDs. The role of exosomal circRNAs in CVDs is summarized and shown in Table 1.

**TABLE 1:** The role of exosomes in CVDs via miRNAs.

miRNA ID	Donor	Recipient	Disease model	Main finding	References
miR-21-3p	Macrophages	VSMCs	AS	Exosomal miR-21-3p from nicotine-treated macrophages may accelerate the development of AS by increasing VSMC migration and proliferation through its target PTEN.	Zhu et al. (2019)
miR-21-5p	Macrophages	Cardiomyocytes	MI	Macrophage exosomes containing miR-21-5p impair cardiac function; aggravate pathology of the myocardial tissue, myocardial fibrosis, and ventricular remodeling; and promote cardiomyocyte apoptosis in MI mice	Dong et al. (2021)
MiR-494-3p	Cardiomyocytes	Myocardial fibroblasts	HF	Peli1 increases miR-494-3p expression in cardiomyocytes during pressure overload-induced HF, and then miR-494-3p is transplanted into myocardial fibroblast via exosomes to promote cardiac fibrosis	Tang et al. (2023a)
miR-21-5p	Cardiac stromal cells from patients with HF	Cardiomyocytes	HF	Intramyocardial injection of exosomes from explant-derived cardiac stromal cells from patients with HF therapy exacerbates cardiac function and left ventricular remodeling via the miR-21-5p/PTEN/AKT pathway	Qiao et al. (2019)
miR-19a-3p	Cardiomyocytes	Endothelial cells	MI	Exosomal miR-19a-3p inhibits endothelial cell proliferation and angiogenesis via targeting HIF-1α and attenuate heart function of mice after MI.	Gou et al. (2020)
miR-155	Cardiomyocytes	Macrophages	Cardiac hypertrophy	HC-derived exosomes activate the MAPK signaling pathway via miR-155	Yu et al. (2020)
miR-512-3p	MSCs	Endothelial cells	AS	MiR-512-3p shuttled by MSC-derived exosomes protect endothelial cells against apoptosis induced by oxidized low-density by targeting Keap1	Chen et al. (2021)
miR-125a-5p	MSCs	Cardiomyocytes	I/R	MiR-125a-5p is enriched in MSC-derived exosomes and exerts obvious cardioprotection via delivery into cardiomyocytes	Gao et al. (2023)
miR-125b-1-3p	Human adipose-derived MSCs	T lymphocytes	AS	MiR-125b-1-3p is expressed in human adipose-derived MSCs and promotes T lymphocyte apoptosis and alleviates AS via downregulating BCL11B expression	Yu et al. (2021)
miR-150-5p	BMSCs	Cardiomyocytes	MI	Exosomal miR-150-5p isolated from BMSCs attenuates apoptosis of cardiomyocytes and improves cardiac function after MI via targeting Bax	Wu et al. (2021)
miR-146a-5p	Cardiosphere	NA	DCM	Improved cardiac function and reduced myocardial fibrosis are noted in animals treated with cardiosphere-derived cell-secreted exosomes depending on miR-146a-5p decreases the expression of TRAF6, Smad4, and FOS.	Hirai et al. (2020)
miR-181a-5p	BMSCs	H9c2 cells	MI	The expression of miR-181a-5p is increased in lipopolysaccharide-stimulated BMSCs. The level of inflammation and oxidative stress can be decreased in H2O2-treated H9c2 cells which are co-cultured with these exosomes via miR-181a-5p targeting ATF2	Liu et al. (2020a)
miR-34a-5p	Macrophages	Cardiomyocytes	ICI-related cardiac dysfunction	Cardiovascular adverse events are normal in patients receiving PD-1 inhibitor treatment. Exosomes derived from PD-1 inhibitor-treated macrophages exerted a pro-senescent effect by modulating the miR-34a-5p /PNUTS signaling pathway	Xia et al. (2020a)
miR-27b-3p	Visceral adipocytes	Vascular endothelial cells	AS	Obesity-induced exosomal miR-27b-3p promotes endothelial inflammation and aggravates AS by targeting PPARα	Tang et al. (2023b)

*PTEN*, phosphatase and tensin homolog deleted on chromosome ten; *HIF-1α,* hypoxia-inducible factor alpha; *BCL11B,* B-cell chronic lymphocytic leukemia/lymphoma 11B gene; *TRAF6* TNF, receptor-associated factor 6; *PNUTS*, serine/threonine-protein phosphatase 1 regulatory subunit 10; *DCM*, dilated cardiomyopathy; *PD-1*, programmed cell death 1; *ICI*, immune checkpoint inhibitor; *PPARα*, peroxisome proliferator-activated receptor α; *NA*, not application.

Previous studies have shown that smoking is strongly associated with AS. But the mechanics are still being explored. Crosstalk between macrophages and vascular smooth muscle cells (VSMCs) via exosomes is essential during the development of AS (Niu et al., 2016). A study suggests that nicotine activates macrophages, leading to the proatherogenic capability of exosomes derived from nicotine-treated macrophages through miR-21-3p (Zhu et al., 2019). Interestingly, miR-21-5p is highly expressed in macrophage-derived exosomes, which contributes to facilitating ventricular remodeling after MI via inhibiting metalloproteinase 3 expression (Dong et al., 2021). Similarly, pressure overload can increase the expression of miR-494-3p in cardiomyocytes, and then intramyocardial delivery into myocardial fibroblast via exosomes, and induce cardiac fibrosis (Tang C. et al., 2023). The exosomal components exhibit enhanced efficacy under pathological conditions. Exosomes isolated from cardiac stromal cells from failing hearts can exacerbate cardiac function and left ventricular remodeling. In contrast, those exosomes isolated from healthy donor hearts result in structural and functional improvement in a murine model of AMI. Qiao group deduced that the difference between the two exosomes in regenerative potential is contributed by miR-21-5p dysregulation which targets the PTEN/Akt pathway (Qiao et al., 2019). In addition, exosomes isolated from cardiomyocytes and hypertrophic cardiomyocytes can also regulate CVDs via miR-19a-3p or miR-155, respectively (Gou et al., 2020; Yu et al., 2020).

Stem cell therapy was once considered a promising treatment strategy for CVDs. But the low efficiency of trans-differentiation into cardiomyocytes after cell engraftment limits this strategy’s therapeutic application. Now, studies have found that stem cell-derived exosomes are more effective and safer than stem cell transplantation in CVD therapy (Yuan et al., 2018). Excessive retention of oxidized low-density lipoprotein in the serum induces endothelial cell apoptosis and then promotes the development of atherosclerosis (Wang C. et al., 2020). In this process, endothelial cells are protected from oxidized low-density lipoprotein overload-induced damage, if they phagocytose mesenchymal stem cell (MSC)-derived exosomes, which are enriched in miR-512-3p (Chen et al., 2021), whereas MSC-derived exosomes can increase cardiac function and limit adverse remodeling after I/R injury, which is dependent on miR-125a-5p (Gao et al., 2023). Exosomes isolated from human adipose-derived MSCs promote T lymphocyte apoptosis and alleviate AS via miR-125b-1-3p (Yu et al., 2021). Bone marrow mesenchymal stem cells (BMSCs), a kind of MSC, have several characteristics, such as easy availability, the powerful capacity of proliferation, and immune-modulatory properties (Guo et al., 2018). BMSC-derived exosomes also exert a regulating role in CVDs. Wu et al. found that BMSC-derived exosomes could facilitate the cardiac function of MI mice via carrying miR-150-5p (Wu et al., 2021). In addition to the abovementioned, cardio-sphere-derived exosomes have also been proven to have a role regulating cardiac function by miRNAs (Hirai et al., 2020). Intracoronary injection of microspheres have been used create a swine model of dilated cardiomyopathy; these models are randomized as preclinical validation of the delivery method and cryosphere-derived cell doses. Through assessing the primary endpoint: safety, and the secondary outcome: change in cardiac function, Hirai et al. discovered that exosome-derived miR-146a-5p from cryosphere-derived cells can mitigate myocardial fibrosis by inhibiting the expressions of pro-inflammatory cytokines and transcripts.

Similarly, the change in the micro-environment makes exosomes have different functions. Using lipopolysaccharide to mimic the inflammatory environment leads to high expression of miR-181a-5p in BMSC-derived exosomes, and these exosomes can alleviate myocardial inflammation and oxidative stress (Liu H. Y. et al., 2020). In addition, there should be a greater exploration of the utility of exosomal miRNAs as significant regulators in immune checkpoint inhibitor-induced cardiovascular adverse events. Downregulation of miR-34a-5p in macrophages can attenuate PD-1 inhibitor-induced pro-senescent effect in cardiomyocytes (Xia et al., 2020a). At the same time, we should also pay attention to cardiometabolic factors, especially with obesity. Tuan’s study found that THP-1 macrophages exposed to interleukin (IL)-4 have a regulatory role in metabolic cardiomyopathy via exosome release (Phu et al., 2022). Their findings show that THP-1-IL4-exosomes can promote mitophagy, mitochondrial activity, and 3T3-L1 adipocyte enlargement via upregulated miR-21/99a/146b/378a and decrease the expression of miR-33 and then control cardiometabolic disease and diabetes in obesity. Another research reveals the positive correlation between obesity and AS (Tang Y. et al., 2023). Tang et al. found that obesity will increase the level of miR-27b-3p in visceral adipose-derived exosomes. This exosomal miR-27b-3p can activate the NF-κB pathway by downregulating PPARα expression in vascular endothelial cells and increasing inflammation and atherogenesis.

### Exosomes serve a regulatory function via lncRNAs

LncRNAs, more than 200 nucleotides in length, rarely have protein-coding potential and are distributed both in endonucleases and cytoplasm. The former could control chromosome modification and inhibit transcriptional activity (Long et al., 2017), while lncRNAs distributed in the cytoplasm could target mRNAs and regulate the expression of the target genes at the transcriptional level (Chu et al., 2015), or inhibit the function of miRNAs by targeting the combination (Beermann et al., 2016). There is a growing body of studies that suggests that it is important in exosomal lncRNAs that we need in the occurrence and progression of diseases. The role of exosomal circRNAs in CVDs is summarized and shown in Table 2.

**TABLE 2:** The role of exosomes in CVDs via lncRNAs.

lncRNA ID	Donor	Recipient	Disease model	Main finding	References
AK139128	Cardiomyocytes	Cardiac fibroblasts	MI	Hypoxia exposure increase the expression of AK139128 in cardiomyocyte-derived exosomes, and these exosomal AK139128 promote apoptosis and inhibit cell proliferation in cardiac fibroblasts after MI.	Wang and Zhang (2020)
MALAT1	Cardiomyocytes	Left ventricular myocardium	MI	Exosomes from cardiomyocytes under HBO at 2.5 atm decrease infarct size and enhance neovascularization via the MALAT1-miR-92a-KLF2/CD31 axis	Shyu et al. (2020)
MALAT1	Human adipose-derived MSCs	Cardiomyocytes	DIC	Cellular senescence is an important influence factor in DIC. Exosomes isolated from human adipose-derived MSCs display pro-metabolism and pro-survival abilities via the MALAT1-miRNA-92a-3p-ATG4α axis	Xia et al. (2020b)
TUG1	Skeletal muscle	Endothelial cells	MI	Exo-lncRNA TUG1 reverses angiogenesis by inhibiting the HIF-1α/VEGF-α axis after MI, and it also maybe a potential marker for MI severity	Dang et al. (2023)
NEAT1	MSCs	Cardiomyocytes	DIC	MIF–treated MSCs protect against Dox-induced cardiac senescence through exosomal NEAT1 targeting to miR-221-3p and leading to Sirt2 activation	Zhuang et al. (2020)
HCP5	BMSCs	Cardiomyocytes	I/R	BMSCs-derived exosomes increase the viability of cardiomyocytes following I/R and decrease apoptosis via HCP5-miR-497- IGF1/PI3K/AKT.	Li et al. (2021)
Mir9-3 hg	BMSCs	Cardiomyocytes	I/R	Exosomal Mir9-3 hg from BMSCs ameliorates I/R injury by inhibiting cardiomyocyte ferroptosis via the Pum2/PRDX6 axis	Zhang et al. (2022a)
KLF3-AS1	MSCs	Cardiomyocytes	MI	KLF3-AS1 in exosomes isolated from MSCs can decrease cell pyroptosis and alleviate MI progression via targeting miR-138-5p to downregulate the expression of Sirt1	Mao et al. (2019)
H19	MSCs	Endothelial cells	AMI	Atorvastatin may play a cardioprotective role after AMI through MSC-derived exosomes via the H19- miR-675-VEGF/ICAM-1 axis	Huang et al. (2020)
ZFAS1	Cardiomyocytes	Cardiac fibroblasts	Chronic kidney disease	In chronic kidney disease condition, ZFAS1 is high expression in cardiomyocyte and –derived exosomes. Exosomal ZFAS1 promotes cardiac fibrosis though the WNT4/β-catenin signaling pathway via targeting miR-4711-5p	Wang et al. (2021)

*KLF2,* Krüppel-like factor 2; *HIF-1α,* hypoxia-inducible factor-1α; *VEGF*, vascular endothelial growth factor; *MIF*, migration inhibitory factor; *IGF1,* insulin-like growth factor 1; *Pum2,* Pumilio2; *PRDX6,* peroxiredoxin 6; *ICAM-1*, intercellular adhesion molecule-1; *DIC*, doxorubicin-induced cardiomyopathy.

Stimulation would influence the normal physiological function of cardiomyocytes, such as hypoxic and hyperbaric oxygen (HBO). By exposure to hypoxic conditions, the expression of lncRNA AK139128, both in cardiomyocytes and cardiomyocyte-derived exosomes, is significantly increased compared with normoxic treatment. Cardiac fibroblasts are co-cultured with exosomes isolated from hypoxic or normoxic-treated cardiomyocytes to simulate intercellular material exchange. Through gain- or loss-of-function experiments *in vitro*, it is confirmed that exosomal AK139128 can stimulate cardiac fibroblast apoptosis and inhibit proliferation, migration, and invasion. It also can exacerbate MI *in vivo* experiments (Wang and Zhang, 2020). Cardiomyocytes are cultured under HBO at 2.5 atmospheres, and exosomes are extracted from these culture cells. Shyu et al. found that HBO significantly increases lncRNA MALAT1 expression both in cardiomyocytes and derived exosomes. Treatment with HBO-induced exosomes can enhance neovascularization and then decrease infarct size after MI via MALAT1-miR-92a-KLF2/CD31 axis (Shyu et al., 2020). Similarly, exosomes circulating after MI carry lncRNA TUG1, which can downregulate angiogenesis by dysfunction of the HIF-1α/VEGF-α axis, and this phenomenon is also confirmed by a randomized controlled trial of post-MI patients, who will receive or not receive percutaneous coronary intervention (Dang et al., 2023).

It is intriguing that MALAT1 is also enriched in exosomes derived from human adipose-derived MSCs following hypoxia treatment. This exosome can inhibit doxorubicin-induced cardiac senescence (Xia et al., 2020b). Similarly, exosomes derived from migration inhibitory factor–treated MSCs protect against doxorubicin-induced cardiac senescence through lncRNA NEAT1 targeting to miR-221-3p (Zhuang et al., 2020). As stated before, MSCs also have positive effects on CVDs via exosomal lncRNAs. LncRNA HCP5 is enriched in human BMSC-derived exosomes and can alleviate myocardial ischemia–reperfusion (I/R) injury via sponging miR-497 to disinhibit IGF1/PI3K/AKT signaling (Li et al., 2021). In parallel, this exosome also contains the lncRNA Mir9-3 host gene (Mir9-3hg) and can attenuate I-/R-induced cardiac injury by regulating ferroptosis (Zhang J. K. et al., 2022). Human MSC-derived exosomes can reduce the MI area, decrease cell apoptosis and pyroptosis, and attenuate MI progression, whereas overexpression of lncRNA KLF3-AS1 in human MSC-derived exosomes via transfection results in significant improvement in the cardiac-protective effects of these exosomes, indicating that human MSC-derived exosomes exert myocardial protection by lncRNA KLF3-AS1 (Mao et al., 2019). Among these studies of MSC-derived exosomes regulating cardiac function, it should be noted that atorvastatin is employed as a lipid-lowering agent, and its heart-protective effect, especially by exosomes, has been recently reported as well (Sun et al., 2021). Atorvastatin pretreatment can increase the expression of lncRNA H19 in MSC-derived exosomes. These H19-overexpression exosomes could promote angiogenesis, migration, and survival of endothelial cells; increase cardiomyocyte survival; decrease fibroblast fibrosis; and ultimately significantly enhance therapeutic efficacy for the treatment of AMI (Huang et al., 2020). The study highlights the fact that a better understanding of the way exosomes are derived from drug-treated cells or organs may provide novel ways to manipulate pathogenesis and the target for drugs.

Many diseases often involve more than a single organ or system, and chronic kidney disease complicated with CVDs is a recognized model (Rafiq et al., 2012; Sarnak et al., 2019). The change in renal function has a positive correlation with the incidence of CVDs (Sarnak et al., 2019), and it is necessary to explore its underlying mechanism. Wang et al. found that the expression of lncRNA ZFAS1 is increasing in the heart and renal tissues of chronic kidney disease mice, and it could be transferred via exosomes from cardiomyocytes to fibroblasts. Exosomal lncRNA ZFAS1 was investigated for its involvement in gene expression modulation to elucidate regulatory mechanisms. It was found to promote cardiac fibrosis through the WNT4/β-catenin signaling pathway, with miR-4711-5p playing a role in this pathway (Wang et al., 2021).

### Exosomes serve a regulatory function via circRNAs

The regulatory role of exosomal circRNAs in CVDs is not random. CircRNAs are primarily produced by exon or intron sequences, and reverse complements or RNA-binding proteins are required for its “life-forms” (Vicens and Westhof, 2014; Zhang et al., 2014; Conn et al., 2015). In addition, its role in CVDs has been confirmed by a series of studies. Accumulating evidence indicates that circRNAs are enriched in exosomes. Exosomes and circRNAs working together can complement each other in regulating CVDs. As previously mentioned, exosomes can serve as platforms of intercellular communications (Bei et al., 2017). Exosomes encapsulate circRNAs and secrete them into peripheral blood, thus protecting them from enzymatic degradation. Then, circRNAs are delivered to target cells and function as competing endogenous RNAs, sponging miRNAs or mRNAs directly, to regulate the expression of target genes, and regulate transcription through RNA Pol II or protein-coding (Ashwal-Fluss et al., 2014; Li et al., 2015; Piwecka et al., 2017). The role of exosomal circRNAs in CVDs is summarized and shown in Table 3.

**TABLE 3:** The role of exosomes in CVDs via circRNAs.

circRNA ID	Donor	Recipient	Disease model	Main finding	References
circRNA-0077930	HUVECs	VSMCs	VSMC senescence	HG exposure increased the expression of circRNA-0077930 in HUVEC-derived exosomes and then induced VSMC senescence	Schick et al. (2015)
circRNA-0006898	Serum	HUVECs	UA	The expression of serum-exosomes circRNA-0006898 was higher in patients with UA, compared with in patients with SA. CircRNA-0006898 promoted the proliferation and migration of HUVECs	Wen et al. (2021)
circRNA-0002113	MSCs	Cardiomyocytes	I/R	CircRNA-0002113 deficiency MSC-derived exosomes alleviate myocardial injury via sponging miR-188-3p to suppress RUNX1 nuclear translocation	Wang et al. (2020c)
has_circ_0097435	Plasma	Cardiomyocytes	HF	Exosomes isolated from plasma blood in HF patients were enriched has_circ_0097435, and it would induce cardiomyocyte apoptosis	Han et al. (2020)
cPWWP2A	Pericytes	ECs	Diabetes	Exosomal cPWWP2A would alleviate diabetic microvascular complications via the crosstalk between pericytes and ECs	Liu et al. (2019a)
circRNA-0001273	UMSCs	Cardiomyocytes	MI	UMSC-derived exosomes were enriched circ-0001273, and exosomal circ-0001273 recovered heart function and reversed cardiomyocyte apoptosis after MI.	Li et al. (2020)
circRNA-0018553	Endothelial progenitor cells	Cardiomyocytes	Cardiac hypertrophy	The expression of circRNA-0018553 is decreased in Ang II-treated cardiomyocytes, but it is enriched in endothelial progenitor cell-derived exosomes. Transplanting these exosomes into cardiomyocytes will alleviate Ang II-induced cardiac hypertrophy via miR-4731 targeting to SIRT2	Sych et al. (2024)
circHIPK3	UMSCs	Myoblasts	MI	UMSC-derived exosomes prevented pyroptosis and enhance ischemic muscle repair by delivering the circHIPK3 via circHIPK3/miR-421/FOXO3a axis	Yan et al. (2020)
	Cardiomyocytes	Cardiac endothelial cells	MI	Hypoxia-pretreatment increased the expression of circHIPK3 in cardiomyocyte-derived exosomes and promoted cardiac endothelial cell migration, proliferation, and tube formation	Wang et al. (2019b)
	Cardiomyocytes	CMVECs	IR	Hypoxia-pretreatment increased the expression of circHIPK3 in cardiomyocyte-derived exosomes and played a protection role in CMVECs	Hewson et al. (2016)
	Umbilical cord MSCs	Cardiomyocytes	HF	Umbilical cord MSC-derived exosomes prevent cardiac senescence via circHIPK3 act as a scaffold to recruit ubiquitin ligase to degrade HuR	Ding et al. (2022)

*HUVECs*, human umbilical vein endothelial cells; *UMSCs*, umbilical cord mesenchymal stem cells; *CMEVCS*, cardiomyocytes-to-cardiac microvascular cells; *EC*s, endothelial cells; *DNMT1* DNA, methyltransferase 1;*SIRT2* sirtuin 2; *UA*, unstable/vulnerable plaque atherosclerosis; *SA*, stable plaque atherosclerosis.

Vascular smooth muscle aging is a leading cause of diabetic complications such as CVDs and kidney diseases or diabetic foot disorders (Chamberlain et al., 2017; Skelley et al., 2018). A previous study has shown that intercellular communication between endothelial cells and VSMCs is critical for the pathogenesis of vascular smooth muscle aging in diabetes (Li S. et al., 2019). By exposing human umbilical vein endothelial cell-derived exosomes to a high-glucose environment, Wang et al. found that they could induce the senescence of VSMCs (Wang S. et al., 2020). Human umbilical vein endothelial cell-derived exosomes exhibit increased abundance of circRNA-0077930 under hyperglycemic conditions. CircRNA-0077930 downregulates the expression of miR-622 and thus increases the expression of Kras, a senescence promoter in vascular cells (Schick et al., 2015). When circRNA-0077930 is exhausted, the exosomes would no longer have the ability to promote VSMC senescence. Carotid plaque rupture and thrombus formation, secondary to unstable/vulnerable plaque AS, is another leading cause of CVDs, such as acute coronary syndrome, ischemic stroke, and peripheral vascular disease (Harari et al., 2019). Yan et al. utilized microarray analysis to demonstrate differential expression of circRNA-0006898 in serum exosomes derived from patients with unstable/vulnerable plaque atherosclerosis (AS) compared to those with stable plaque AS. This suggests a potential involvement of exosomal circRNA-0006898 in plaque instability. By gain- or loss-of-function experiments, they clarify that upregulation of the expression of circRNA-0006896 in serum-exosomes from patients with unstable/vulnerable plaque AS induces the proliferation and migration of human umbilical vein endothelial cells, depending on the circRNA-0006896–miR-1264-DNMT1 axis (Wen et al., 2021).

In shedding light on I/R injury, people pay more attention not only to the damage to heart function but also to microcirculation dysfunction (Yu et al., 2018). By exchanging oxygen and metabolites between blood and cardiomyocytes (Hewson et al., 2016), the microcirculation may be significant to protect microvasculature from I/R injury. Wang et al. have found an important exosome that plays an essential role in cardiomyocyte-to-cardiac microvascular cell communication under I/R (Wang et al., 2019b). The research by Tian et al. also enriches the theoretical basis of exosome therapy for I/R injury (Tian et al., 2021). Silencing circRNA-0002113 through siRNA treatment in MSC-derived exosomes reduces cellular apoptosis in an anoxia-reoxygenation model, thereby mitigating ischemia/reperfusion (I/R) injury through the miR-188-3p/RUNX1 axis. The expression of circHIPK3 in exosomes derived from hypoxia-pretreated cardiomyocytes was upregulated. This exosomal circRNA is transferred into cardiac microvascular cells and induces the protection of oxidative injury by targeting miR-29a. It is noteworthy that they also find these hypoxic exosomes can reduce the infarct area and promote the angiogenesis infraction border zone after MI via a similar mechanism (Wang Y. et al., 2020). CircHIPK3 has been demonstrated to be involved in regulating apoptosis, proliferation, migration, and angiogenesis (Li Y. et al., 2017; Shan et al., 2017; Liu N. et al., 2018; Liu X. et al., 2018), but has not been associated with exosomes before. This finding suggests whether the previously discovered circRNAs may play new regulatory roles by shuttling from cells to cells via exosomes. In addition, microvascular stabilization is often disrupted by diabetes. Pericyte-derived exosomes which load circRNA cPWWP2A regulate endothelial cell biology under diabetes-related stress to maintain a stable state via the crosstalk between vascular pericytes and endothelial cells (Liu C. et al., 2019).

Li et al. provide new insights into the repair of ischemic injury. After MI, the expression of circ-0001273 is downregulated and then recovered, indicating that circRNA-0001273 might be a significant regulator of ischemic injury. They find a positive correlation between the expression of circRNA-0001273 in the exosomes and in the human umbilical cord MSCs (hucMSCs). *In vivo* experiments demonstrated that intracardiac injection of 100 μL of exosomes derived from hucMSCs significantly improved left ventricular ejection fraction (LVEF) and left ventricular fractional shortening (LVFS) in MI rat models. Notably, this therapeutic effect was absent in control groups receiving either PBS or exosomes isolated from hucMSCs transfected with si-circRNA-0001273, which exhibited impaired secretory capacity following RNA interference treatment (Li et al., 2020). Coincidentally, circHIPK3 is also detected in hucMSC-derived exosomes. Yan et al. found that hucMSC-derived exosomes could prevent pyroptosis and enhance ischemic muscle repair by delivering circHIPK3, which can inhibit inflammation by targeting miR-421 (Yan et al., 2020). HF is attributed to many CVDs such as cardiac hypertrophy, myocardial fibrosis, and ischemic cardiomyopathy. Combined with a next-generation sequencing executed on five HF patients and four health controls with a quantitative polymerase chain reaction analysis performed on 40 HF patients to make a comprehensive judgment, the expression of has_circRNA-0097435 is confirmed to be significantly upregulated in HF patients. Overexpression of has_circRNA-0097435 could induce cardiomyocyte apoptosis, while silencing of has_circ_0097435 would reverse this process. Notably, the study also finds the expression of has_circRNA-0097435 in exosomes extracted from plasma blood is significantly increased in patients with HF (Han et al., 2020). Although the function of exosomal has_circRNA-0097435 is not clear yet, there is speculation that exosomes enriched in has_circRNA-0097435 are transferred into other cardiomyocytes and then promoted in HF. In another study, circHIPK3 is confirmed as a scaffold for HuR and β-TrCP. CircHIP3 deficiency could attenuate the interaction of HuR with β-TrCP and decreases HuR ubiquitination, promoting the interaction of HuR with p21 mRNA. Ding et al. demonstrated that cardiomyocyte-specific circHIPK3 knockout mice exhibit worse cardiac function with the increasing expression of the senescence-inducer p21. In addition, the senescence phenotype could be reversed by umbilical cord MSC-derived exosomes through delivering circHIPK3 (Ding et al., 2022). Zuo et al. showed a novel regulatory mechanism of cardiac hypertrophy (Zuo et al., 2023). Endothelial progenitor cell-derived exosomes can replenish the deficiency of circRNA-0018553, which is decreased expression in Ang II-treated cardiomyocytes, and then attenuate cardiac hypertrophy by targeting miR-4731. It may also be useful in the prevention of HF. These findings enrich the possibilities of exosomal circRNAs as a therapeutic tool for CVDs.

### Exosomes serve as biomarkers of CVDs via ncRNAs

In addition to being applied clinically, exosomes also have indicative functions in diseases (Kalluri and LeBleu, 2020). The degree of extracellular secretion also depends on specific cellular micro-environment and external factors, such as cellular stress and activation signals (Zhang et al., 2015). Therefore, detecting the cargo of exosomes could reflect the changing state of their original cells and could potentially serve as diagnostic biomarkers for CVDs (Liu et al., 2021). Especially, ncRNAs act as cargos. Exosomal ncRNAs exhibit better stability compared with ncRNAs distributed in plasma or cytoplasm. They are potent biomarkers as they can be easily detected in body fluids (blood, urine, etc.) due to their remarkable stability and are present in exosomes (O'Brien et al., 2018).

The miRNAs have natural properties, making them excellent potential clinical biomarkers. For example, exosomal miR-92a-3p released by endothelial cells is associated with arteriosclerosis and may be a potential diagnostic biomarker (Liu Y. et al., 2019). Moreover, the current European guidelines emphasize the urgent need for the use of miRNAs as biomarkers to guide clinical decision-making in HF and shift the clinical decision core from conventional care to personalized and precision medicine (Ponikowski et al., 2016; Bayes-Genis et al., 2018).

The function of identifying lncRNAs released for extracellular and intercellular communication is limited in comparison with miRNAs. Notably, extracellular lncRNAs have inferior properties. Detecting the expression of lncRNAs in exosomes, which are isolated from the plasma of AMI patients and healthy controls via sequencing profiles and twice qRT-PCR validations, Zheng et al. found that the two lncRNAs, ENST00000556899.1 and ENST00000575985.1, are elevated in AMI patients with top fold change compared with 518 differentially expressed lncRNAs (Zheng et al., 2020). Beyond that, the results of the ROC curve and multivariate logistic model analysis also indicate an association of the two lncRNAs with the risk of HF in AMI and can function as potential biomarkers for predicting AMI prognosis. Nonetheless, the application of lncRNAs as HF biomarkers in the future is definite. LncRNAs are stable and less affected by the microenvironment and can be detected in extracellular fluids. The relatively low expression of lncRNAs may be resolved by performing PCR to amplify the sequences.

Previous studies have proved four advantages of circRNAs as biomarkers: 1) excellent stability depending on their circularized structure; 2) widespread distribution; 3) the content of circRNAs in the blood is higher than that of other ncRNAs; 4) hundreds of cell-specific circRNAs have been identified and can be the candidates of CVD biomarkers (Sun et al., 2020). But for exosomal circRNAs, the fifth advantage is that circRNAs can be encapsulated by exosomes to prevent enzymatic degradation. Thus, there is evidence that exosomal circRNAs are potential biomarkers for CVDs. RNA sequencing is used to analyze the expression of circRNAs in exosomes isolated from the plasma samples from three coronary artery disease patients and three paired controls. From 164 upregulated circRNAs and 191 downregulated circRNAs, has_circ_0005540 is selected as a candidate biomarker with significant expression differentiation (fold change > 4, P < 0.05), higher association with CVDs (P < 0.0001), and obvious discriminatory power in ROC analyses (AUC = 0.853, 95% CI = 0.799–0.906, P < 0.001) [160]. As HF is irreversible, a sensitive, accurate, and specific biomarker can help predict the risk of HF during the progression of CVDs. Exosomal has_circ_0097435 might be a better one. Its expression is known to increase in the exosomes of HF patients’ peripheral blood compared with healthy patients, implying an indicative function (Han et al., 2020). Using oxidized low-density lipoprotein (ox-LDL) to treat HUVECs to induce AS, studies confirm that both circRNA-0026218 and circRNA-0004104 can inhibit the proliferation of HUVECs and promote apoptosis by targeting different miRNAs/target proteins axis, respectively (Zhang Y. et al., 2022; Liu et al., 2024b). Importantly, the expression of the two circRNAs is upregulated in exosomes isolated from ox-LDL-treated HUVECs. Similarly, circRNA-0086296 promoting atherosclerotic lesions via the IFIT1/STAT1 feedback loop by sponging miR-576-3p has been proven (Zhang M. et al., 2022). However, the highlight of this study is that Zhang et al. found increasing expression of circ-0086296 in exosomes of patients with AS and derived by ox-LDL-treated ECs. It means exosomal circRNA-0086296, circRNA-0026218, and circRNA-0004104 may act as collaborative indicators of AS, which may have better sensitivity and accuracy.

### Intercellular delivery systems and potential application of exosomes

The cellular system has been metaphorically likened to a bio-factory capable of manufacturing diverse molecular cargoes for extracellular transport via EVs. Among EV subtypes, exosomes serve as specialized couriers in intercellular communication. Current drug-loading strategies primarily utilize two approaches: (1) pre-secretory loading through parental cell-derived therapeutic agents and (2) post-secretory modification employing electroporation or sonication techniques (Zhang et al., 2020). Beyond their paracrine functions, exosomes that can play the role of systemic regulatory potential through hematogenous dissemination to distant cellular targets have been demonstrated (Rayner and Hennessy, 2013). Target cells participate in initiating cargo discharge through membrane fusion or endocytosis, completing intercellular substance delivery. A major attraction of exosomes as therapeutic delivery vehicles lies in their intrinsic advantages over synthetic delivery systems such as liposomes, nanoparticles, or polymeric micelles (Sych et al., 2024). These benefits include superior biocompatibility, minimized immunological side-effects, effective evasion of rapid clearance through macrophages, and efficient penetration across biological barriers (such as the blood–brain barrier). Additionally, exosomes spontaneously utilize native cell surface recognition molecules (e.g., integrins, tetraspanins, and glycoproteins) for preferential targeting to specific cell types, tissues, or organs, providing the foundation for personalized, precision medicine (Su et al., 2024).

By harnessing or engineering exosomes, researchers can load specific therapeutic agents to treat diseases. For instance, exosomes isolated from mesenchymal stem cells (MSC-Exosomes) have been widely investigated in regenerative medicine due to their capability to deliver anti-inflammatory cytokines, proangiogenic factors, and miRNAs associated with tissue regeneration and repair (Ardalan et al., 2021). Numerous studies demonstrate the therapeutic potential of MSC-Exosomes in treatment of myocardial infarction, chronic heart failure, osteoarthritis, and even liver fibrosis, where they significantly decrease inflammation, increase angiogenesis, and enhance cellular regeneration by transferring specific miRNAs and growth factors (Lin et al., 2022). In oncology, exosomes have tremendous potential for targeted chemotherapy and immunotherapy. Engineered exosomes can carry therapeutic agents such as chemotherapeutic drugs (e.g., doxorubicin and paclitaxel), as well as nucleic acid-based therapeutics (e.g., miRNAs, siRNAs, and CRISPR components), for targeted anticancer therapy (Zhang et al., 2023). Engineered exosomes that display tumor-specific targeting moieties (such as anti-HER2 antibodies) or pH-sensitive release properties can accumulate preferentially in tumor tissues, significantly improving the therapeutic efficacy over conventional chemotherapeutic agents while reducing off-target toxicity substantially. In neurological disorders, exosomes exhibit remarkable potential owing to their intrinsic ability to cross the blood–brain barrier, enabling targeted delivery of therapeutic biomolecules to the central nervous system. Initial studies have efficiently utilized exosomes to deliver neuroprotective factors such as BDNF, GDNF, and curcumin, successfully ameliorating pathological symptoms in Alzheimer’s and Parkinson’s disease models (Iyaswamy et al., 2023). Moreover, the exosomal delivery of therapeutic miRNAs shows encouraging results in stroke recovery, suggesting their potential in clinical management of acute neurological injuries. This endogenous transport mechanism has spurred significant therapeutic advances, particularly for RNA-based therapies that require protection from serum nucleases and effective cell membrane penetration. Lipid-based delivery systems, utilizing their biomimetic membrane compatibility, have become promising RNA carriers (Tibbitt et al., 2016; Kaczmarek et al., 2017). Clinical validation comes from patisiran, an siRNA therapeutic agent approved by the US Food and Drug Administration, which uses lipid nanoparticles (LNPs) to treat hereditary transthyretin-mediated amyloidosis (Coelho et al., 2020).

Exo-Fect, a nucleic acid transfection reagent developed by SystemBiosciences, has been recently found to directly transfect nucleic acids, including siRNAs, miRNAs, mRNAs, and even plasmid DNA, into isolated exosomes. The central problem of how specific cargo is loaded into exosomes is solved by simply combining nucleic acid substances with Exo-Fect reagents according to the operating instructions. Exosomes can carry these nucleic acids into target cells, which also provides a new possibility for exosomes to be used in clinical therapy. The transfection efficiency with Exo-Fect reagents may be significant compared to the *in vitro* manipulation experiments we have used previously, such as electroporation, heat shock, detergent-based compound (saponin) penetration, or cholesterol-modified miRNAs. Ricardo et al. demonstrated that miR-55-5p could be transferred to endothelial cells via sEVs and perform its function using the Exo-Fect reagent. More interestingly, they found that the membrane of the EXO-Effect-loaded EVs was altered compared to the natural EVs, which enhanced the internalization of the EXO-Effect-loaded EVs within the target cells and reduced the interaction of these regulated EVs with lysosomes (de Abreu et al., 2021). The research results of Chen et al. also prove this result. EVs derived from adipose mesenchymal stem cells isolated using a 3D culture platform composed of porous gelatin methacryloyl are rich in has-miR-455-3p. This miRNA can promote hyaline cartilage regeneration by activating the PAK2/Smad2/3 axis. Compared with engineering optimization strategies such as agomir/lentivirus transfection and electroporation, Exo-Fect treatment achieved relatively better transfection efficiency (Chen J. G. et al., 2024). These studies suggest that it is feasible to play a role through engineered modification of exosomes from parental cells, including the delivery of specified nucleic acids into exosomes by transfection, which are then delivered to the target cell. It is even possible to imagine that we can remove the substance from exosomes and then transfect the required nucleic acids into the empty exosomes, thereby reducing the possible side effects.

Despite significant progress, there are still key challenges in achieving precise sorting of extracellular vesicle molecules. A major issue involves developing strategies for selectively encapsulating therapeutic ingredients while eliminating potential harmful components. Heteronuclear ribonucleoprotein A2B1 (hnRNPA2B1) was identified as a key RNA-binding mediator through RNA pull-down analysis, demonstrating its specific interaction with lncARSR transcripts in a sunitinib-resistant renal cell carcinoma model. It is worth noting that neither siRNA-mediated inhibition of hnRNPA2B1 nor overexpression of lncARSR variants with mutated hnRNPA2B1-binding domains increased the level of extracellular vesicle lncARSR, ultimately confirming the important role of hnRNPA2B2 in sequence-specific RNA packaging [169]. Garc í a-Mart í n et al. supplemented these findings (Garcia-Martin et al., 2022). The determinants of miRNA sorting were systematically characterized through biophysical and proteomic analyses. Their work revealed two key selection criteria: sequence features: miRNAs rich in exons exhibit higher G + C content and higher Gibbs free energy. The dual layer sorting mechanism of RNA-binding proteins Alyref and Fus coordinating sequence recognition and vesicle transport provides physical, chemical, and protein-mediated specificity. Mechanism understanding enables us to improve delivery efficiency through engineered miRNA sequences, regulate targeted tissue distribution through circulating miRNAs, and use endogenous sorting pathways for therapeutic disease intervention. These molecular gatekeeping systems fundamentally enhance our ability to manipulate extracellular vesicle cargo components for precision medicine applications.

Second, the biological challenge of targeted delivery efficiency of extracellular vesicles. The non-specific distribution of extracellular vesicles entering the circulatory system poses a significant technical challenge. The liver and kidneys, as the main clearing organs, often have significantly higher drug and RNA concentrations than other tissues. When the therapeutic target is not these two organs, off-target toxicity may occur. The existing solutions include the following: (1) optimizing the route of administration, achieving local targeting through intratumoral injection or nasal administration (Mizrak et al., 2013; Sterzenbach et al., 2017); (2) surface engineering modification, introducing targeted ligands to enhance the specific recognition ability (Zhang et al., 2020). It is worth noting that only 0.7% of extracellular vesicles can reach the target organ after intravenous injection, highlighting the urgency of improving the delivery system.

Third, receptor cell uptake mechanism and efficiency optimization. Extracellular vesicles deliver their contents through multiple pathways such as membrane fusion, endocytosis, and surface ligand binding. Among them, membrane fusion pathway relies on lipid raft domains, integrins, and micro-environmental pH values (Levy and Shoham, 2005; Parolini et al., 2009; Valapala and Vishwanatha, 2011; Mulcahy et al., 2014) to directly release the contents into the cytoplasm. The efficiency of the endocytosis pathway covering various clathrin-mediated, lipid raft-mediated, and caveolin-mediated mechanisms (Gurung et al., 2021) is significantly affected by particle size (<100 nm particle uptake rate increased by 3.5 times) (Caponnetto et al., 2017). The signal transduction pathway is involved in mediating immune regulation and apoptosis regulation through surface ligand–receptor interactions (Munich et al., 2012; Sobo-Vujanovic et al., 2014). There is cross-regulation of different endocytosis mechanisms: the clathrin and caveolin pathways share regulatory molecules (Gurung et al., 2021), while macropinocytosis ultimately leads to lysosomal degradation (Kerr et al., 2006; Gordon, 2016). The temperature sensitivity (5.8 times higher uptake efficiency at 37°C compared to 4°C) suggests that the influence of the dynamic micro-environment in the body needs to be considered (Escrevente et al., 2011).

**FIGURE 5:**
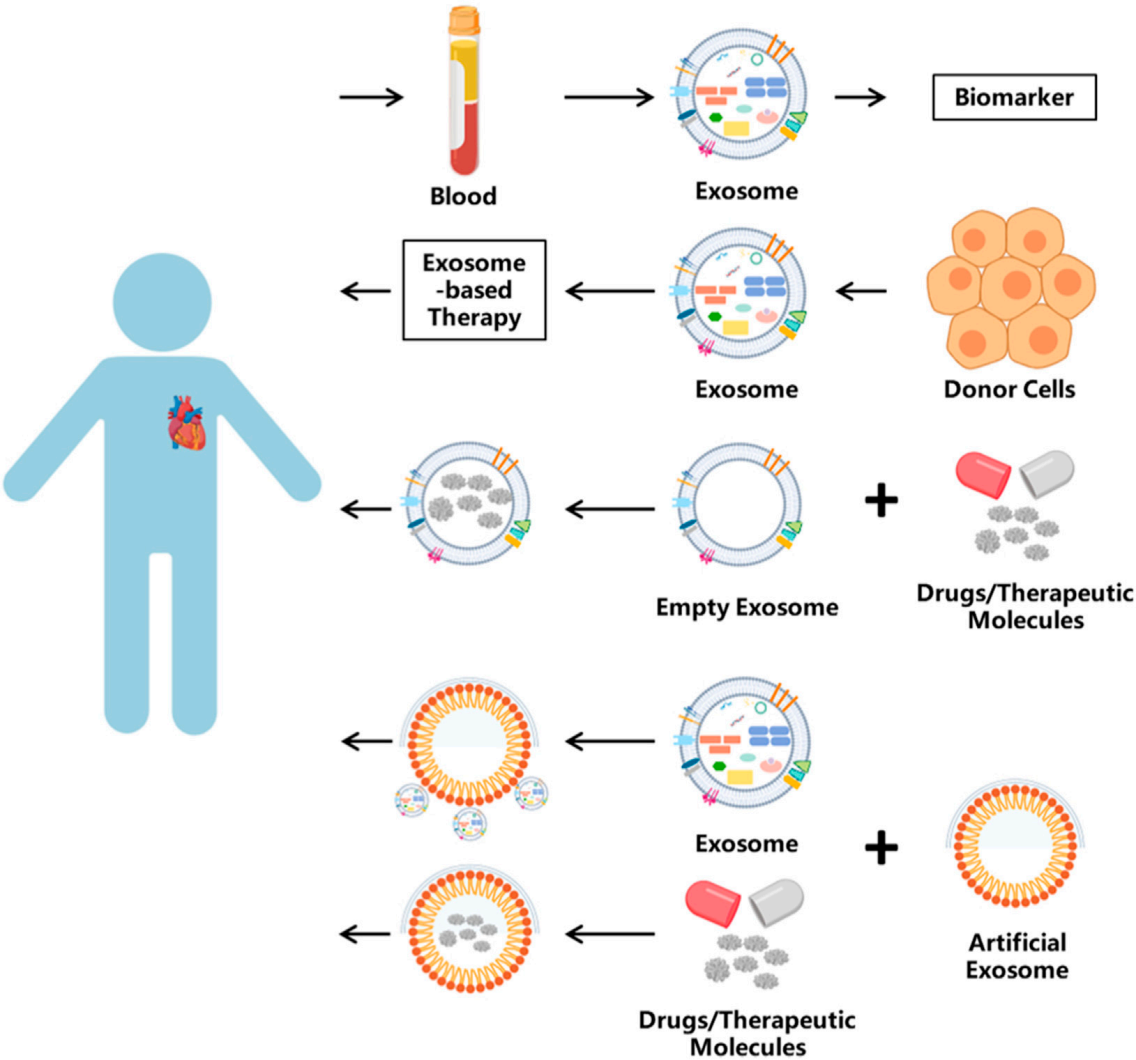
The application of exosomes. Exosomes have many uses. One is used as biomarkers, which are isolated from blood or other body fluids. Exosome-based therapy has been applied in clinical treatment. Or as the vehicle, exosomes can carry bioactive molecules into target cells.

Now, clinical studies on extracellular vesicles have been widely conducted, and Table 4 lists the active CVD-related trials registered on ClinicalTrials.gov. However, the clinical translation of natural extracellular vesicles is still limited by these unresolved challenges. Engineering exosomes have become a promising alternative, utilizing their innate ability as intercellular delivery carriers to encapsulate various therapeutic payloads while maintaining physiological functions (Samir et al., 2013; Pegtel and Gould, 2019) Strategic modifications, including ligand coupling and surface functionalization, significantly improved their targeting accuracy and transfection efficiency (Zhan et al., 2020). Unlike engineering modification, artificial exosomes have practical application value. It is worth noting that endogenous circulating exosomes exhibit cardioprotective effects after myocardial infarction (MI) (Cheng et al., 2019), and their limited homing to ischemic areas (<2% cumulative efficiency) hinders therapeutic efficacy. Liu et al. created a nanoparticle composed of Fe_3_O_4_ core and silica shell, which can capture extracellular vesicles under local magnetic fields and acidic pH microenvironments. In the myocardial infarction model, circulating extracellular vesicles captured by nanoparticles accumulate around the infarcted tissue under magnetic guidance, leading to a reduction in the myocardial infarction area and an increase in left ventricular ejection fraction (Liu S. et al., 2020). Meanwhile, micro/nano motor systems represent a paradigm shift in active drug delivery. These self-propelled devices convert chemical, optical, or electrical energy into directed motion (Wang, 2009), and their adaptive control mechanisms enable them to navigate in the pathophysiological microenvironment (Li T. et al., 2017). Their integration with extracellular vesicles creates a hybrid platform capable of autonomous targeting and payload release, potentially revolutionizing the accuracy of intracellular delivery. A breakthrough application involves bioengineered EVs for protein recovery therapy. You et al. demonstrated that in an aging skin model, EV-mediated delivery of COL1A1 mRNA restored skin collagen synthesis by 68%, establishing a blueprint for the treatment of age-related protein deficiencies (You et al., 2023). Furthermore, tumor-derived exosomes can reshape the tumor microenvironment through the transmission of oncogenic signals, promoting tumor growth, angiogenesis, immune evasion, and metastasis (Chen S. et al., 2024). In neural systems, neuron- and glial cell-derived exosomes are involved in synaptic plasticity, nerve regeneration, and modulation of neuro-inflammation by shuttling neurotrophic factors, synaptic proteins, and regulatory miRNAs across distinct cell types (Xian et al., 2022). Collectively, these unique physiological functions render exosomes an attractive candidate for controlled intercellular communication and intervention, offering previously unattainable therapeutic possibilities. This strategy is particularly promising for situations involving low expression or mutated proteins, as shown in the system overview of exosome engineering applications in Figure 5.

**TABLE 4:** The projects of exosomes in CVDs registered in ClinicalTrials.gov.

ID	Study type	Donor	Conditions/disease	Interventions/treatment	Purpose of applying exosomes
NCT04127591	Observational	Peripheral blood	MI	Collect exosomes in peripheral blood of patients and extract miRNAs from them	Collect exosomes in peripheral blood of patients with MI, compare the expression of miRNA with healthy volunteers, find out the miRNAs with significant differences, and explore its relationship with the development of MI.
NCT03478410	Interventional	Epicardial fat	AF	Epicardial fat biopsy	(1) Compared with healthy controls, whether epicardial fat in patients with AF release quantitatively and qualitatively different exosomes (2) Whether epicardial fat-derived exosomes can be biomarkers, treatment and prevention targets for arrhythmias
NCT04142138	Interventional	Urine	Prehypertension	DASH diet which is based on low salt and high potassium components	To characterize changes in urine electrolytes and exosome protein abundance pattern during nutritional changes, exploring the effectiveness of DASH diet in lowering blood pressure
NCT03034265	Observational	Urine	Hypertension	NA	Determine the concentrations and variabilities of urinary exosomal sodium channel protein in patients with difficult-to-treat arterial hypertension and to identify whether it can be a new biomarker for the classification and monitoring of difficult-to-treat arterial hypertension
NCT02822131	Interventional	Urine	Hypertension	(1) Low phosphate: sevelamer sodium bicarbonate sodium chloride; (2) high phosphate: sodium phosphate	Assess changes in the NCC activity as the mechanism of phosphate-sensitive blood pressure regulation through detecting changes in sodium/chloride-cotransporter NCC and NaPi-IIa from urinary exosomes
NCT03984006	Observational	Urine	Thyroid disease/HF	NA	Detect the urine exosomal proteins NT-proBNP in patients with subclinical hyperthyroidism or subclinical hypothyroidism, and try to find out the correlation biomarker for heart dysfunction in autoimmune thyroid disease
NCT03660683	Interventional	Urine	Type 2 diabetes/cardiovascular diseases	(1) Dapagliflozin 10 mg (2) Saxagliptin 5 mg (3) Placebo oral tablet	Urine exosome assay to evaluate the cardioprotective of effect of the saxagliptin and dapagliflozin combination on endothelial progenitor cells in patients With type 2 diabetes
NCT03837470	Interventional	Urine	HFpEF	(1) 0.9% sodium chloride (2) Furosemide 40 mg	Sodium transporters in urinary exosomes will be characterized and compared between HFpEF patients and controls to evaluate renal sodium excretion after salt loading in HFpEF.
NCT04334603	Interventional	Plasma	HF	Exercise training	Plasma exosomes will be isolated and detected to assess the impact of exercise training on established surrogate markers in HF.

*AF*, atrial fibrillation; *DASH*, dietary approach to stop hypertension; *NCC*, NaCl co-transporter, *NaPi-IIa*, sodium phosphate co-transporter IIa, *HFpEF*, heart failure with preserved ejection fraction; *NA* not application.

## Conclusion

In this review, we systematically delineate the dual biological and therapeutic significance of exosomes in cardiovascular pathophysiology. As naturally evolved nanoscale communicators, exosomes exhibit sophisticated cargo-sorting mechanisms governed by RNA-binding proteins (e.g., hnRNPA2B1) and sequence-specific thermodynamics, enabling precision regulation of intercellular signaling networks. Their endogenous secretion pathways and membrane fusogenic properties position exosomes as paradigm-shifting vectors in cardiovascular therapeutics, demonstrating remarkable potential in myocardial repair, angiogenesis modulation, and atherosclerosis regression. Current clinical applications leverage exosomes’ innate cardio-protective properties through stem cell-derived vesicles, while emerging engineering strategies—including magnetic nanoparticle guidance, ligand-directed targeting, and synthetic exosome mimetics—address critical challenges in spatiotemporal delivery control and payload efficiency. Nevertheless, translational acceleration requires deeper mechanistic insights into exosome biogenesis heterogeneity and recipient cell processing dynamics. Future research directions should focus on the following: (1) multi-omics characterization of cardiovascular disease-specific exosomal signatures; (2) development of stimulus-responsive “smart exosomes” with microenvironment-adaptive release profiles; (3) standardization of GMP-compliant manufacturing protocols for clinical-grade production; (4) integration with artificial intelligence-driven delivery optimization platforms.

As we unravel the intricate code of exosome-mediated cross-talk, these biological nanoparticles are poised to bridge the critical gap between fundamental cardiovascular research and precision medicine implementation, ultimately redefining therapeutic paradigms for complex CVDs.

## Abbreviations

CVDs, cardiovascular diseases; HF, heart failure; AMI, acute myocardial infarction; AS, atherosclerosis; I/R, ischemia reperfusion; HBO, hypoxic and hyperbaric oxygen; EVs, extracellular vesicles; MVBs, multi-vesicular bodies; ILVs, intraluminal vesicles; VSMCs, vascular smooth muscle cells; MSCs, mesenchymal stem cells; BMSCs, bone marrow mesenchymal stem cells; ncRNAs, non-coding RNAs; miRNAs, microRNAs; lncRNAs, long non-coding RNAs; circRNAs, circular RNAs; TSG101, tumor susceptibility gene 101; ESCRT, endosomal sorting complex required for transport; IL, interleukin; Ox-LDL, oxidized low-density lipoprotein; hnRNPA2B1, heterogeneous nuclear ribonucleoprotein A2B1; COL1A1, extracellular-matrix α1 type-I collagen.
